# Interaction between the leg recovery test and subjective measures of fatigue in handball players: short-, mid-, and long-term assessment

**DOI:** 10.3389/fspor.2024.1474385

**Published:** 2024-12-19

**Authors:** Julian Bauer, Thomas Muehlbauer, Sheila Geiger, Markus Gruber

**Affiliations:** ^1^ Department of Sport Science, Human Performance Research Centre, University of Konstanz, Konstanz, Germany; ^2^ Division of Movement and Training Sciences/Biomechanics of Sport, University of Duisburg-Essen, Essen, Germany; ^3^ Clinic for Psychosomatic Medicine and Psychotherapy, LVR-University Hospital Essen, University of Duisburg-Essen, Essen, Germany; ^4^ Centre for Translational Neuro- and Behavioral Sciences (C-TNBS), University of Duisburg-Essen, Essen, Germany

**Keywords:** athlete self-report measures, exhaustion, monitoring, regeneration, team sports, workload

## Abstract

**Background:**

The physical and mental demands of handball during training or competition often lead to fatigue which can impair performance. Many attempts have been made to assess the level of fatigue in athletes either by objective (neuromuscular performance) or subjective (questionnaires) measures, however, their interplay over short-, mid-, and long-term periods is currently unknown. Knowledge about both types of assessments is important as load management by coaches is traditionally based on direct adjustments following a training session, adjustments of content structure of training weeks between games, as well as adjustments of load management over the entire competitive season. Thus, this study aimed to investigate the interplay between objective and subjective fatigue measures at multiple test times throughout a handball season.

**Methods:**

A total of 100 highly trained (Tier level 3) adolescent or young adult team handball players (23 females) took part in the study. The parameters tested were the Leg Recovery Test (LRT score) which is based on the countermovement jump height (CMJ) and was assessed by a commercial wristwatch (Polar Vantage V2) as an objective measure of neuromuscular fatigue. Additionally, on a subjective level, questionnaire-based athlete self-report measures, specifically the Perceived Recovery Status Scale (PRSS) and the Short Scale of Recovery and Strain (KEB) were assessed. We used non-parametric tests to detect differences between relevant test time points (short-term: immediately following one handball-specific training session, i.e., from T_0_ to T_1_; mid-term: over the course of three consecutive training days, i.e., from T_0_ to T_2_; long-term: over the course of 8 months of training, i.e., from T_0_ to T_12_) and linear mixed models to evaluate the interplay between objective (LRT score) and subjective (KEB score and PRSS score) measures of fatigue across one season.

**Results:**

Non-parametric tests showed that CMJ height (*p* = .012) and the KEB (*p* < .001) were higher at T_1_ compared to T_0_ for the short-term assessment. Over the course of three consecutive training days (i.e., mid-term assessment), the CMJ height score decreased (T_0_ to T_2_: *p* < .001; T_1_ to T_2_: *p* = .018) and the PRSS score (T_0_ to T_2_: *p* < .001; T_1_ to T_2_: *p* = .003) increased. Linear mixed models revealed no significant effects of KEB or PRSS score on LRT score (i.e., CMJ height) for the short- and mid-term assessments. In terms of the long-term assessments, we detected no general direct or interaction effects of PRSS score, workload, and test time point on LRT score, except for an interaction between PRSS score and workload on LRT score (*p* = .032), which indicates a workload-dependent association between PRSS and the objective fatigue measure (LRT score).

**Conclusion:**

Athlete self-reported measures of fatigue indicated significantly higher cumulative fatigue after both short- and mid-term periods, whereas this increase was observed in the LRT score only during the mid-term period. Furthermore, the absence of a relationship between the objective and subjective measures of fatigue during short- and mid-term periods suggests that these measures assess distinct types of fatigue. In the long-term assessments, the significant interaction between the PRSS score and workload on the LRT score suggests that higher workloads are associated with an increased correlation between subjective (PRSS score) and objective (LRT score) measures of fatigue. This indicates that perceived fatigue may be a more sensitive indicator of fatigue, which can be managed to maintain high levels of neuromuscular performance (LRT score). However, with higher workloads (>10 h per week), associations between the objective and subjective measures become apparent, suggesting that workload serves as a common factor influencing overall fatigue.

## 1 Introduction

In team sports, many clubs invest substantial resources in monitoring the load of their athletes (1). Thereby, one of the greatest challenges in managing training and match load (i.e., workload) is the fact that these stressors are applied on a team level. However, sport teams are a collection of individuals and the psycho-physical responses to the workload differ significantly between individual athletes (2) leading to different strains and subsequently different levels of fatigue in the players. Consequently, monitoring fatigue plays a crucial role in planning the training stimuli over different training periods (3). More importantly, it must be distinguished between external (input or stimulus) and internal (individual response) load assessments. Many of the newer monitoring technologies such as GPS-tracking, power output, speed, accelerometry, or time-motion analysis focus on the measurement of external load. The increasing availability of these data may lead coaches to solely focus on such external load measurements (2). However, it has been suggested that the internal processing of this load should be given a higher priority than the external stimuli (4), making individual assessments desirable. A variety of parameters, such as heart rate, lactate, or VO_2_max, are used to monitor the internal load (5). Yet, some of these measurements are challenging to perform during continuous training and games, especially in team sports. One such team sport that is widely played, especially in Europe, is handball (6). In handball, various studies and reviews have analysed the physical demands and internal load, particularly during competition. For example, a recent review by García-Sánchez et al. (7) reported the total distance covered during matches at a national level to be 4,506.7 ± 647.9 m while the running pace differed between female (110.5 ± 7.2 m/min) and male players (78.4 ± 19.7 m/min) during competition. Moreover, backcourt players were reported to perform more throws than pivots and wings while pivots exhibited more body contact than the other playing positions. A study by Michalsik (8) compared the on-court actions between playing positions but also between offence and defence. The author reported varying numbers of actions in terms of playing time (min), breakthroughs, fast breaks, technical errors, tackles, claspings, screenings and shots between offence and defence. Additionally, the author concluded that the demands placed on the player's aerobic system are moderate to high as indicated by a mean relative workload of approximately 70%–80% of VO_2_max during match-play. Additionally, moderate to high post-match blood lactate values were observed in male players indicating a substantial effort of the anaerobic energy systems. These were also reported in another study (9) which found that mean post-match blood lactate concentration (BLC) was 4.8 mmol/L for male players with large inter-individual differences (2.8–10.8 mmol/L). Additionally, Wagner et al. (10) concluded in their review that team handball players require a high level of aerobic capacity to regenerate during low-intensity periods, thereby ensuring their ability to perform at high-intensity phases of the game (VO_2_max of 55–60 ml/kg/min; BLC peak 8–12 mmol/L) throughout the entire 60 min of a match. Additionally, Wagner et al. (10) concluded a relative workload of 65%–80% of VO_2_max, a total distance per match of 3,900–4,700 m, a mean heart rate (HR) of 160–170 beats/min, a high number of activity changes (600–1,500 per match), and a post-match BLC of 3–11 mmol/L. Another parameter that has been used more frequently is metabolic power (11). Metabolic power does not only focus on pure running performance, but also considers the varying metabolic work during accelerations, which are more demanding than simply maintaining speed (12). Furthermore, it also considers the impact of body contact actions and collisions.

Due to the myriads of test procedures practitioners, who want to implement monitoring methods in the field must decide which tests are most suitable. For the utilization of a long-term continuous monitoring procedure, it is crucial that the tests are highly practical, inexpensive, time efficient, well accepted by coaches and athletes and easy to interpret (13). Furthermore, the tests should not physically interfere (3), disrupt the training schedule (14), and provide the relevant data at short notice (15). Additionally, for a parameter to be considered a valid indicator of an athlete's fatigue, it must be sensitive to both training and competition load (16).

For the coach and practitioner working in the field, however, it is not only the type of assessment, which is important, but also the timing of the respective measurements. Based on the distinction between different training cycles (17), three specific points in time or time periods are crucial for coaches and athletes. Firstly, the immediate response to a training stimulus (*short-term assessment*). Secondly, the effects of a specific training week (*mid-term assessment*), i.e., adaptation and recovery after a match followed by preparation for the next match. This period, often called microcycle, represents a particularly difficult challenge, as it normally takes place between two competitions, i.e., matches, and coaches must therefore ensure adequate recovery while simultaneously prepare their athletes for the following match. Thirdly, over an entire competitive season (macrocycle), with its typical progression of alternating phases of recovery and adaptation to the high number of matches with the goal to assess chronic cumulative load adaptation (*long-term assessment*). The importance of assessing fatigue at different times results from the fact that recovery and stress are influenced by various factors and intervals.

Regardless of the time of testing, fatigue results from external stimuli and it can be differentiated between performance (objective) fatigue and perceived (subjective) fatigue, as suggested by Kluger et al. (18). Furthermore, Behrens et al. (19) classified fatigue into *motor performance fatigue*, *perceived motor fatigue*, *cognitive performance fatigue*, and *perceived cognitive fatigue*. Fatigue in sports can be tested on three different pathways: (a) performance-oriented, for example by using jump height as the objective reference measure, (b) questionnaires, often referred to as ASRM (athlete self-report measures) based on the subjective self-assessment of the athlete, and (c) biomarkers, which provide an objective measure of the biochemical and physiological processes taking place in the body (20). Biomarkers have the advantage of being objective, accurate, and reproducible but are also associated with great effort and expense (21). A more practical way to test the neuromuscular fatigue of athletes is the countermovement jump (CMJ), which is often considered as the gold standard (22). CMJ height is an objective performance parameter which has been frequently used to draw conclusions on the current fatigue or state of recovery of an athlete. A meta-analysis by Claudino et al. (23) has confirmed the high validity of CMJ height as an internal individual indicator of neuromuscular fatigue with the average CMJ height of multiple jumps exhibiting greater sensitivity compared to the highest CMJ height. However, determining CMJ height using force plates is often not feasible or available for in field measurements. An alternative is the measurement of CMJ height by a commercially available sports-watch, the *Polar Vantage V2*. It provides the *leg recovery test* (LRT) which is integrated into the software of the watch and measures the average jump height of three successive CMJs. This test has been recently validated using force plate data (24). The major advantages of the LRT are that it only requires athletes to perform a limited number of jumps (i.e., three) and that it is non-invasive. Thus, it is very time-efficient and puts only limited stress on the athletes. Moreover, it can be used at the training site and thus exclude the potentially confounding influence of an artificial laboratory-based assessment (25).

It is well documented that fatigue cannot only limit physiological (e.g., jump performance) but also cognitive processes (22). One way to include psycho-physical aspects are psychometric tests (ASRM). Psychometric methods usually ask the athletes for their perceived physical condition, state of fatigue and recovery. Laurent et al. (26) describe ASRM as psychobiological tools that also assess life load (i.e., sleep, nutrition, life stress, etc.). There are several validated ASRM to assess athlete's fatigue status. Meyer et al. (27) rated psychometric procedures as the most successful measure for assessing fatigue. Especially in larger groups, such as team sports, psychometric methods can be effectively employed due to their efficiency in time and cost (28). However, questionnaires can be rather time-consuming when answering all the items. To counteract this, adapted and shorter questionnaires are frequently used in team sports (3). Based on the differentiation of Behrens et al. (19), questionnaire items represent a *subjective perceived fatigue indicator* while the LRT can be regarded as an *objective performance fatigue indicator*. Bourdon et al. (15) suggest that combining both measures of fatigue over an extended period to evaluate training effectiveness and adjust programs may be beneficial as the coach and practitioner should not only be concerned with the athlete's performance and potential fatigue but also with their perceived physical condition.

In contrast to soccer, which has vastly been studied in terms of monitoring the athletes’ load (29), there has been limited research on monitoring fatigue in handball to date. This is particularly disadvantageous as it has been increasingly recognized that the playing positions in handball have distinctive requirement profiles and diverging loading patterns (30). Only one study evaluated the sensitivity of both the CMJ and ASRM items as an indicator of fatigue in handball players. Buchheit (31) examined amongst others the sensitivity of psychometric measures and CMJs to detect fatigue in highly trained adolescent handball players immediately before a training session. The category *fatigue* in the POMS (profile of mood state questionnaire) did not correlate with CMJ height. Thus, the author concluded that using questionnaires to gauge fatigue cannot accurately predict changes in the physical performance of highly trained adolescent handball players. It should be noted that these assessments were conducted at specific times on the same day of the week (Mondays) every 2–4 weeks during the competition period, thus immediate pre-/post-assessments following a training or match stimulus or assessments during consecutive days of a training week were not part of their assessments.

In sum, no study in handball differentiates between the distinct assessment times relevant for coaches and athletes, i.e., immediately after a training session (*short-term*), over the course of consecutive training days (*mid-term*), and over the course of a competitive season (*long-term*). Due to this lack of studies and the novelty of the LRT, the aim of the present study was to investigate the interplay between the different testing procedures (LRT and ASRM) in handball players. This can help practitioners or coaches to decide which test to use to monitor an athlete's fatigue. If for instance, significant correlations between the two measures exist, it would be sufficient to solely use one of the tests, whereas low correlations could indicate that the tests assess different types of fatigue and should therefore be used in combination. We hypothesized that (1) the LRT performance (i.e., CMJ height) will decrease and ASRM (KEB and PRSS scores) will increase following handball-specific workload; (2) we will find correlations between decreases in LRT scores and increases in KEB and PRSS scores after handball specific short-, mid-, and long-term assessments.

## 2 Methods

### 2.1 Study design

**Figure 1:**
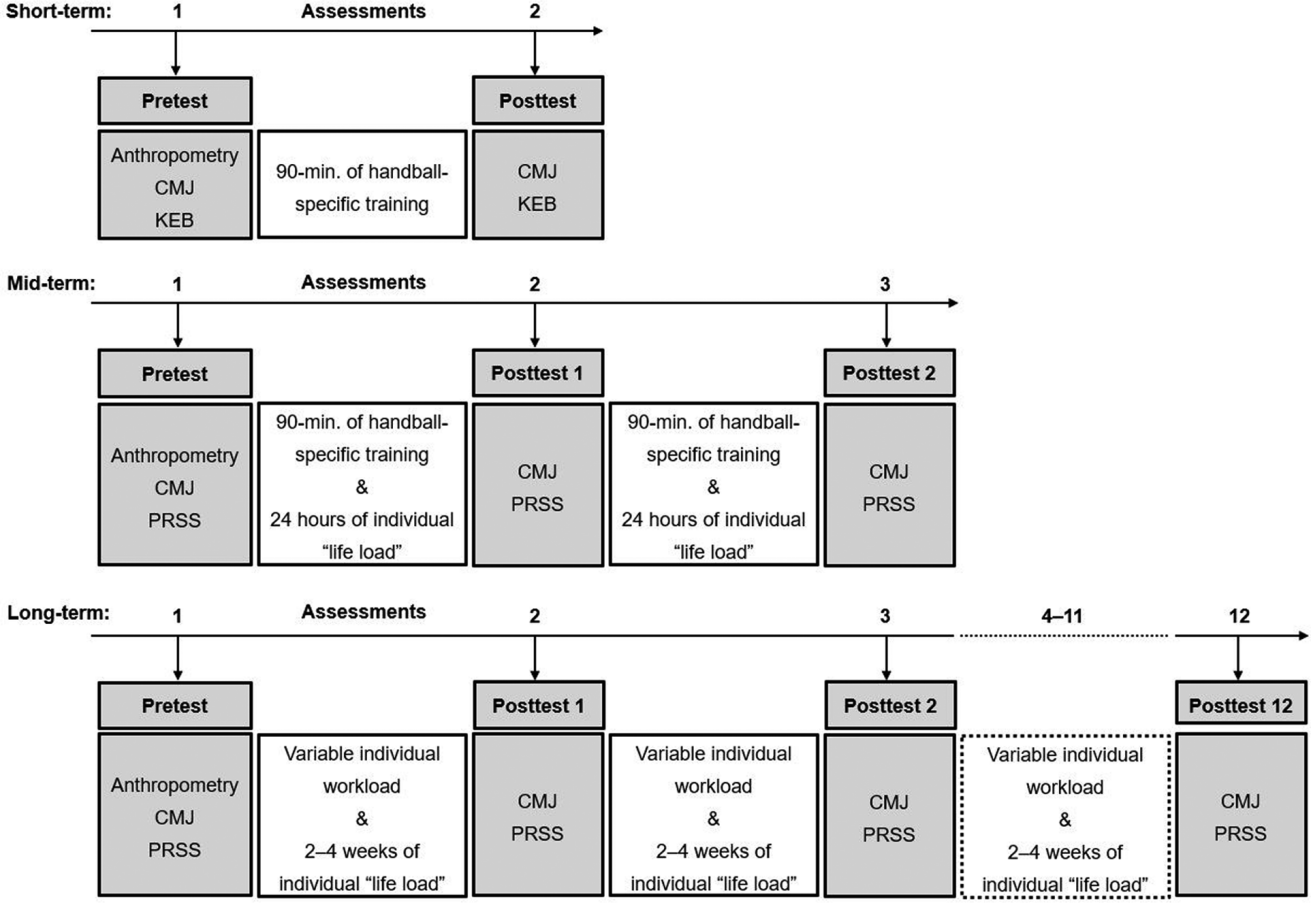
Schematic diagram of the study designs. CMJ, countermovement jump; KEB, short scale of recovery and strain; PRSS, perceived recovery status scale.

The study employed a repeated measures design, distinguishing between three different durations and reference times (Figure 1). *Short-term* (i.e., study 1): pre-/post-evaluation took place before (T_0_) and immediately after a handball-specific (90 min.) training stimulus (T_1_). *Mid-term* (i.e., study 2): evaluation was carried out at T_0_ (before the first session on training day 1), at T_1_ (before the second training session on training day 2, i.e., 24 h later), and at T_2_ (before the third training session on training day 3, i.e., 48 h after T_0_). *Long-term* (i.e., study 3) assessments were conducted before each training session from the first (T_0_) to the last training session over a period of 8 months (T_0_–T_12_). The training sessions of the long-term evaluation took place every 2–4 weeks, depending on public and school holidays. All assessments (short-, mid-, and long-term) were conducted on Monday evenings (or started then in the case of the mid-term assessments) to eliminate the influence of the time of day.

### 2.2 Participants

All participants were highly trained handball players (Tier level 3) according to the classification system of McKay et al. (32) and had a handball training frequency of 3–5 sessions plus one match per week. Further characteristics of the players can be found in Table 1. Twelve of the players took part in both the mid- and long-term assessments. To be included in the long-term analysis, participants were required to have actively participated in at least 80% of the training sessions. Participants who could not take part in the respective training session of the testing day or those who reported any musculoskeletal, neurological, or orthopedic disorder within the preceding 4 weeks of the testing procedure were excluded for the assessment of this training. Written informed consent was obtained from the participants’ parents or legal guardians (in case they were under the age of 18), and participants also gave their assent. The study protocol adhered to the Declaration of Helsinki for human experimentation and the ethics standards of the University of Konstanz. The study protocol was approved by the University of Konstanz's ethics committee (reference number: IRB24KN011-02w).

**Table 1:** Participant characteristics by assessment.

Variable	Short-term assessment	Mid-term assessment	Long-term assessment
Teams (*n*)	3	2	1
Sample size (*n*)	48	25	27
Sex (female; *n*)	10	13	0
Age (years)	15.7 ± 1.7	19.0 ± 5.0	15.5 ± 1.3
Body height (m)	1.77 ± 0.09	1.75 ± 0.08	1.73 ± 0.11
Body mass (kg)	71.1 ± 12.1	70.2 ± 16.5	74.2 ± 12.3
Body mass index (kg/m^2^)	24.2 ± 3.2	22.1 ± 2.1	22.3 ± 2.2
Training experience (years)	9.4 ± 3.6	10.1 ± 3.1	8.2 ± 2.7

Data are group mean values ± standard deviations.

### 2.3 Testing procedures

The Leg Recovery Test (LRT), KEB (Short Scale of Recovery and Strain), and anthropometry of the *short-term* assessment were carried out immediately before the training session after a short and standardized warm-up (which was also executed before the mid- and long-term assessments). The standardized warm-up comprised 2 min of submaximal running and three submaximal countermovement jumps (CMJs) to familiarise the participants with the target movement. This was followed by a handball-specific 90-min training session which, in accordance with the framework training concept of the German Handball Federation, consisted of a preparatory general part (small games and exercises), a handball-specific part (basic games for attack and defence) and a final handball game. Immediately after the training session, the surrogate parameters (LRT and KEB) were measured again and recorded for further statistical analysis. The *mid-term* assessment started with the testing of the LRT, Perceived Recovery Status Scale (PRSS), and anthropometry immediately before the first training session of the first training day at the beginning of the training week. This was followed by a 90-min training session with the same training content as during the short-term assessment. The next tests (LRT and PRSS) took place 24 h after the first tests, i.e., immediately before the second training session on training day two, which always took place at the same time in the evening. This was followed by another 90-min training session with similar training content as the previous sessions, and the third tests (LRT and PRSS) on training day 3 which took place again 24 h later, before the third training session. The *long-term* assessment process began with the tests (LRT, PRSS, anthropometry) again immediately before the first training session on the first training day at the beginning of the training week. This was followed by a 90-min training session with comparable training content as the other sessions and the training and match load (i.e., workload of 2–4 weeks), which differed between the players. The next tests (LRT and PRSS) took place before the next training session of this team, which trained at an interval of 2 weeks (always on the same day of the week, i.e., Mondays at the same time in the evening), so that both the life load and the (self-reported) sport-specific workload were considered. This interval of tests T_0_ to T_12_ before the next training session (LRT and PRSS) was continued over a period of 8 months, except public holidays. All tests took place during the competition period.

### 2.4 Anthropometric measurements

In the case of the short-term assessments, all participants were asked by one of the testers to self-report their body height, body mass, age, gender, and training experience. For the mid-, and long-term assessments participants were asked to stand up straight and without shoes while their body height was measured using a Seca 217 linear measurement scale (Seca, Basel, Switzerland) to the nearest 0.1 cm. Participants wore light clothing and no shoes when their body mass was measured using an 803 electronic scale (Seca, Basel, Switzerland) to the closest 100 g. By dividing each participant's body mass by their squared body height (kg/m^2^), the body mass index was determined. Additionally, every participant provided their years of training experience.

### 2.5 Leg recovery test

In accordance with the recommendations of the German Handball Federation (33), a standardized warm-up (see chapter 2.3) including typical running and mobility exercises was performed by each participant. Afterwards, one of the testers demonstrated the execution of the LRT to all participants. All CMJs, i.e., the LRT jumps, were executed in line with the procedure and descriptions of Bosco et al. (34). In preparation for the jumps, participants had to place their hands on their hips while standing in an upright position with their legs nearly straight. Vibration and sound signals were given by the watch before each of the three jumps. The participants were required to commence the exercise by squatting as rapidly as possible, with the knees bent to approximately 90°. This was followed by a simultaneous, maximal dynamic straightening of both legs, with the objective of jumping as high as possible. During the flight phase, the legs had to be held straight (knee angle: 180°) and they were only allowed to be bend immediately before touchdown to ensure a gentle landing. Holding the arms akimbo was required throughout the entirety of the jumps. The inertial measuring unit integrated in the wristwatch (Polar Vantage V2) calculated the mean jump height (cm) of all three jumps (LRT-score). Using the mean jump height of repetitive trials is recommended, as research analyzing CMJ measurements demonstrated that the average jump height of CMJs provides more reliable information regarding an athlete's neuromuscular status compared to the highest jump (23). In a preceding study, the LRT of the Polar Vantage V2 sports watch has been validated and showed a mean error of ≈5% and a high correlation (*r* = 0.96; *p* < 0.001) for the jump height compared to force plate data (24). Moreover, the authors reported high day-to-day reliability (*r* = 0.91; ICC = 0.79).

### 2.6 Assessment of subjective (perceived) fatigue

Two different questionnaires were used, as the time intervals of the measurement could not be represented equally well by both selected questionnaires (KEB and PRSS). The *Short Scale of Recovery and Strain* (KEB) is a questionnaire which was introduced to meet the need for a valid, economical, and change-sensitive measurement instrument for the acute state of recovery and stress in sport (35). The preceding training stimulus may have an almost exclusive influence on the assessment immediately after exercise (postexercise evaluation) and it was therefore used for the short-term assessment. One strength of the questionnaire is its practical applicability in training monitoring, as it is well suited to the needs of applied sports practice due to its compact, yet multidimensional, valid, and sensitive form (35). The item “*general state of stress*—*for example, exhausted, weakened, overloaded, physically exhausted*” was used as the reference item (on a scale of 0–6: with 0 equating to no fatigue and 6 equating to highly fatigued). In contrast, the *Perceived Recovery Status Scale* (PRSS) (26) also considers life load factors (diet, sleep habits, etc.) and recovery times between training sessions and matches, which could affect both mid- and long-term fatigue. The PRSS requires a specific recovery interval to determine the status of the athlete after a training or competition stimulus, which makes it rather unsuitable for immediate post-training assessments. The PRSS is a psychobiological tool to identify fatigue based on a 0–10 scale (with 0 equating very poorly recovered/extremely tired and 10 equating very well recovered/highly energetic). Therefore, the KEB was used for the short-term assessments while the PRSS was used for the mid- and long-term assessments. Both questionnaires allowed half numbers to be selected, i.e., 1.5 or 4.5, etc. All subjects were asked to rate their perceived level of recovery before the training and, in the case of the acute response to training (short-term fatigue), also immediately after training. According to Laurent et al. (26), the PRSS reveals a decline in performance among fatigued athletes, particularly at the highest scale values. To avoid confusion with the KEB scale which indicates fatigue by starting with 0 = no fatigue and 6 = highly fatigued, the PRSS was used in a reversed version with 0 indicating very well recovered and 10 indicating very poorly recovered/extremely tired.

### 2.7 Statistical analyses

Data analysis was conducted using SPSS Statistics 26 (IBM) and R 4.3.3 (R Core Team). Shapiro-Wilk test was conducted to assess the normal distribution of the outcome measures (i.e., LRT, KEB, and PRSS score) during the short- and mid-term assessments. As the data was not equally distributed in both cases, Wilcoxon signed rank tests for the short-term assessments and Friedman's ANOVA for the mid-term assessments were conducted to examine significant differences between the individual testing times using *z*-scores, *χ^2^*-scores, and *T*-scores as test statistics. Pearson's correlation coefficients were calculated to determine effect size (*ES*). Following Cohen's interpretation guidelines, *ES* = .10, .30, or .50 signifies a small, moderate, or large effect (36). Further, linear mixed models (LMM) were conducted to assess the relationship between objective and subjective measures of fatigue across the different test time points, as they allow to deal with both fixed and random effects (37). Fixed effects in the model for the short- and mid-term assessments included subjective fatigue scores and time points, along with their interaction terms to explore potential moderating effects of test time points on the relationship between subjective and objective fatigue measurements. For the long-term assessments, fixed effects in the model involved subjective fatigue scores, training workload, and test time points, along with their interaction terms. Participant variable was used as a random effect to account for between-subject variability. Model assumptions were verified through residual diagnostics (homoscedasticity, normality, linearity, and random intercepts normal distribution). To enhance the robustness and reliability of our model estimations, we utilized the mixed function from the afex package (38). Significance was set at an alpha level of 0.05, with degrees of freedom approximated using Satterthwaite's method. The analyses were performed using the lme4 package for linear mixed models (using the function lmer), the lmerTest package for a comprehensive depiction of results, and the afex package for advanced model handling (39). *A priori* power analyses were conducted using G*Power (40) for within-subject ANOVA, targeting a small-to-moderate effect size (ηp^2^ = 0.06) and a power of 0.8. This required sample sizes of 34 for the short-term and 28 for the mid-term assessments, which we met through successful recruitment. For the long-term assessments with 13 measurements, a sample of 12 participants was sufficient for adequate power. Although we recruited 27 participants, missing data was frequent, a common issue in longitudinal studies. This motivated our switch to a mixed linear model approach, which accommodates incomplete cases.

## 3 Results

### 3.1 Measures of fatigue during short- and mid-term assessments

Table 2 shows the descriptive statistics (M ± SD) for the objective and subjective measures of fatigue by assessment. In terms of *short-term assessment*, the LRT score was significantly higher (*z* = −2.51, *p* = .012, ES = .26) in T_1_ than in T_0_. Regarding subjective measures, the KEB score was significantly higher (*z* = −4.46, *p* < .001, ES = .46) in T_1_ than in T_0_. Concerning *mid-term assessment*, LRT scores significantly changed (*χ^2^* = 16.29, *p* < .001) across test time points. Pairwise comparisons using Wilcoxon signed rank tests with adjusted *p*-values showed that LRT scores significantly decreased from T_0_ to T_2_ (*T* = 1.07, *p* < .001, ES = .42) and from T_1_ to T_2_ (*T* = 0.37, *p* = .018, ES = .15). Further, the PRSS scores significantly changed (*χ^2^* = 21.86, *p* < .001) over the course of three consecutive training days. Pairwise comparisons yielded significant increases from T_0_ to T_2_ (*T* = −1.23, *p* < .001, ES = .50) and from T_1_ to T_2_ (*T* = −0.87, *p* = .003, ES = .34).

**Table 2:** Descriptive statistics for LRT (i.e., countermovement jump height) and subjective (i.e., KEB and PRSS scores) measures of fatigue by assessment.

Test	Short-term assessment		Mid-term assessment		Long-term assessment	
	LRT	KEB	LRT	PRSS	LRT	PRSS
T_0_	27.9 ± 7.4 [25.7:30.0]	2.3 ± 1.3 [1.91:2.68]	31.5 ± 4.9 [29.5:33.5]	3.6 ± 2.4 [2.62:4.58]	39.3 ± 4.8 [37.4:41.2]	2.0 ± 1.1 [1.55:2.45]
T_1_	29.2 ± 7.1 [27.1:31.3]^a^	3.4 ± 1.0 [3.13:3.70]^aaa^	30.8 ± 5.2 [28.7:33.0]	4.8 ± 1.8 [4.01:5.51]	35.6 ± 4.8 [33.7:37.5]	2.5 ± 1.3 [2.00:3.00]
T_2_	–	–	29.4 ± 5.0 [27.4:31.5]^aaa^	6.4 ± 1.4 [5.85:7.02]^aaa^	37.7 ± 6.4 [35.2:40.2]	2.1 ± 1.4 [1.56:2.68]
T_3_	–	–	–	–	39.2 ± 6.0 [36.9:41.6]	2.9 ± 2.4 [1.92:3.81]
T_4_	–	–	–	–	38.0 ± 5.2 [35.9:40.0]	5.3 ± 2.7 [4.28:6.38]
T_5_	–	–	–	–	37.8 ± 5.5 [35.7:40.0]	4.2 ± 2.8 [3.06:5.30]
T_6_	–	–	–	–	34.1 ± 5.4 [32.0:36.3]	3.9 ± 1.9 [3.14:4.64]
T_7_	–	–	–	–	38.2 ± 5.8 [36.0:40.5]	4.2 ± 2.3 [3.31:5.10]
T_8_	–	–	–	–	37.8 ± 5.1 [35.8:39.8]	2.4 ± 1.9 [1.68:3.16]
T_9_	–	–	–	–	38.5 ± 5.3 [36.4:40.5]	3.9 ± 1.5 [3.36:4.53]
T_10_	–	–	–	–	38.7 ± 5.3 [36.6:40.8]	3.2 ± 1.4 [2.67:3.80]
T_11_	–	–	–	–	38.2 ± 5.5 [36.1:40.4]	3.4 ± 2.5 [2.47:4.41]
T_12_	–	–	–	–	35.1 ± 4.0 [33.5:36.7]	3.6 ± 2.5 [2.61:4.59]

Data are group mean values ± standard deviations. Values in brackets indicate 95%-confidence intervals. LRT, leg recovery test score (i.e., CMJ height in cm); KEB, short scale of recovery and strain (0–6: with 0 equating no fatigue and 6 equating highly fatigued); PRSS, perceived recovery status scale (0–10: with 10 equating very well recovered/highly energetic and 0 equating very poorly recovered/extremely tired). Superscript a indicates significant differences from t0 in the Wilcoxon signed rank test (for short-term assessment) or the *post hoc* test subsequent to the initial Friedman test (for mid-term assessment), and superscript b indicates significant differences from t1 in the respective *post hoc* test.

^a^*p* < 0.05.

^aaa^*p* < 0.001.

### 3.2 Interaction between LRT and subjective measures of fatigue during short-, mid-, and long-term assessments

**Figure 2:**
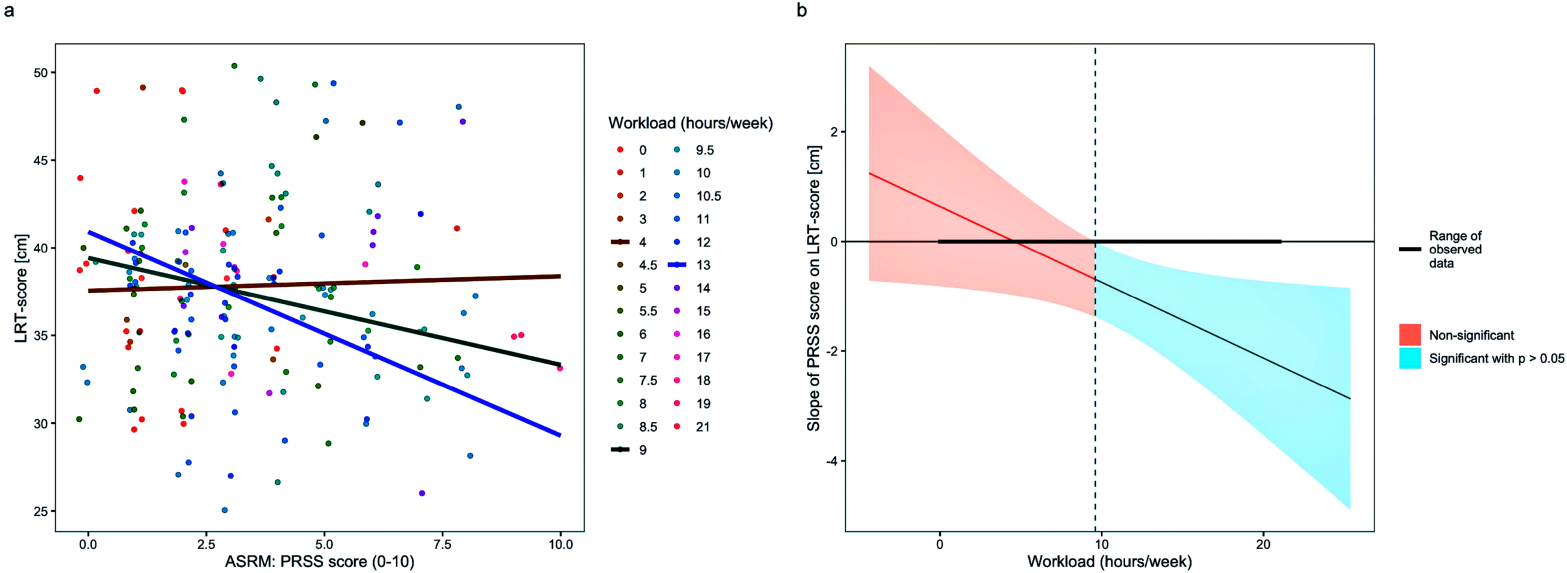
Interaction between athlete self-report measures (ASRM) and leg recovery test (LRT) performance. **(a)** Jitterplot of perceived recovery status scale (PRSS) score (0–10) and workload (hours/week) on countermovement jump (CMJ) height. Dots represents jitters of individual data points in terms of the relationship between the LRT score and the PRSS score. The lines represent marginal effects from the mixed linear models reported above. The three exemplary marginal slopes illustrate the interaction between workload and PRSS on LRT score during 4, 9, and 13 h per week. The points do not model the raw data, but the mixed linear model. The marginal slopes are selected as examples for better visualization. **(b)** Association between the slope of the relationship between the PRSS and workload on LRT scores with Johnson-Neyman intervals. Significant deviances from a slope of 0 occur around a workload of 10 h per week. This interaction might be interpreted by assuming that at lower workload there is apparently no relationship between PRSS and the LRT. With higher workload we see an increasingly pronounced negative relationship between PRSS and the LRT.

All calculated models met the key assumptions meaning that residuals demonstrated homoscedasticity and normality, with no linearity violations observed and random intercepts mostly follow a normal distribution. In terms of *short-term assessment*, the LMM analysis did not reveal significant direct effects of KEB score [*F_(1, 57.73)_* = 0.08, *p* = .785] or test time point [*F_(1, 49.02)_* = 0.01, *p* = .908] on LRT score. The interaction between KEB score and LRT score from T_0_ to T_1_, did not reach statistical significance [*F_(1, 48.03)_* = 0.40, *p* = .529]. Concerning *mid-term assessment*, the LMM analysis did not reveal significant direct effects of PRSS scores [*F_(1, 50.55)_* = 0.07, *p* = .787], but of test time points [*F_(2, 44.99)_* = 3.70, *p* = .033] on LRT scores. The interaction between PRSS scores and LRT scores across test time points did not show statistical significance [*F_(2, 44.90)_* = 2.34, *p* = .108]. Regarding *long-term assessment*, the LMM analysis did not reveal significant direct effects of PRSS scores [*F_(1, 111.64)_* = .74, *p* = .390], workload [*F_(1, 113.54)_* = 3.55, *p* = .062] or test time points [*F_(12, 112.42)_* = 1.48, *p* = .143] on LRT scores. However, there was evidence for a significant interaction between PRSS scores and workload [*F_(1, 112.42)_* = 4.74, *p* = .032] on LRT scores. This interaction was explored using simple slope analyses as implemented in the R packages *emmeans* (41) and *interactions* (42). Figure 2a depicts three exemplary slopes or associations between PRSS and LRT scores at different workloads. Figure 2b shows the association between the slope of PRSS on LRT scores and weekly workloads. The corresponding calculations suggest that the relationship deviates statistically from a straight line—i.e., no correlation—starting from a workload of about 10. Further interaction terms did not reach statistical significance [PRSS scores and test time point, *F_[12, 113.26]_* = 1.47, *p* = .144; workload and test time points, *F_[12, 112.30]_* = 1.28, *p* = .238; PRSS scores and workload and test time points, *F_[12, 113.07]_* = 1.27, *p* = .244].

## 4 Discussion

This study is the first to investigate three highly relevant time periods for the assessment of fatigue following handball-specific workload in highly trained (Tier level 3) adolescent or young adult team handball players. The first period was the immediate postexercise response after a single training session (short-term assessment). The second period (microcycle) was the influence of a specific training week on the development of fatigue (mid-term assessment). The third period (macrocycle) was the entire season, during which there is long-term adaptation and recovery after a match, followed by preparation for the next match (i.e., accumulated training and match load) over the whole competitive season (long-term assessment). In this way, the analysis considered both performance (objective) and perceived (subjective) fatigue over the three distinct time periods.

### 4.1 Differences in objective and subjective measures of fatigue during short- and mid-term assessment

In line with the first hypothesis stating that ASRM will increase, and LRT performance will decrease following handball-specific workload, we were able to show a higher perceived fatigue in the KEB scores directly after one handball training session (i.e., short-term assessment). However, in contrast to our assumption, we detected an increase but not a decrease in the LRT score. For mid-term assessments, the aforementioned hypothesis was fully confirmed as the ASRM increased and the LRT scores decreased over the duration of three consecutive training days. The finding of higher jump height in the LRT in the short-term assessment seems counterintuitive at first glance and stands in contrast to the findings of (43–46) as well as (47) who all reported decreases in CMJ height immediately after one handball-specific training session. Several reasons may be responsible for the lack of the expected decreases in CMJ height (i.e., LRT scores) in the short-term. The enhanced jump height observed directly following the handball training session may be attributed to a more efficient activation of the muscles of the leg extensors immediately after handball training than after the warm-up due to frequent jumps during training (48). Therefore, possible fatigue may have been overcompensated for by a better “preparedness” for the jumps. This explanation also is in line with Thorlund et al. (44) who proposed a change in jumping strategy following handball-specific workload to avoid performance decreases which may have led to increases in our cohort. Another possible explanation for the lack of the expected LRT score decrease could be that lactate and ammonium which have been proposed as being responsible for performance decreases in jumps (49), both were unable to exert their short-term detrimental effects immediately after the handball-specific training load. Furthermore, the updated framework on fatigue and human performance proposed by Behrens et al. (19) appears to indicate that the potential adverse effects on motor performance fatigue were not present during the LRT. The mean HR during handball matches was reported to be approximately 85% of the maximum HR (HR_max_), as observed by Kniubaite et al. (50) and Manchado et al. (51). Similarly, Wagner and Hinz (52) stated that the relative workload of elite male players during matches is approximately 70%–80% of the VO_2_max or 55–60 ml/kg/min, as reported by Wagner et al. (53). However, Póvoas et al. (45), Michalsik et al. (9) and Türkmen (54) reported lactate levels of around 2–3 mmol/L post-competition, which merely differ from the resting lactate levels (55) and are therefore unlikely to be a factor in performance reduction. This also corroborates the capacity of handball players to recover between high-intensity periods (as during training sessions and matches), a hypothesis posited by Michalsik (8), Martínez-Rodríguez et al. (56) and Pluncevic Gligoroska (57). Therefore, as far as the possible detrimental influence of these biomarkers is concerned it should also be noted that in team sports training such as handball with its intermittent interval characteristic (58), there are always phases of low exertion for the players, which may help to remove various metabolic by-products that may have an acute detrimental influence on the LRT scores after the training session. In line with this thought, the contractile functions of the leg extensors, which enable the realization of the LRT, may have not yet been restricted immediately after the end of training as metabolic by-products may not have exert their detrimental influence so soon after the training (59). Another reason for the improved LRT scores immediately after the training session could be the phenomenon of post-activation performance enhancement, which refers to an improvement in strength, power, or speed following voluntary contractions (60, 61). Similar mechanisms were also described in young handball players by Dello Iacono et al. (62) and Al Kitani et al. (63) who both reported increased jump heights following different kinds of jumps during handball training sessions. This may have also been the case in our sample as the handball-specific training vastly includes reactive jumping performance (for example during jump shots and blocks) which is similar to the demands of LRT. Apart from the above-mentioned arguments, the inconsistent results (increased vs. decreased jump height after one handball training session) may also result from different intensities and loads during the training sessions of the studies. Perhaps, performance-positive effects of the training session compete with performance-negative effects and together they produce the observed inconsistent results. Thus, the intensity and load of the training session with its specific characteristics may play a crucial role when investigating fatigue by LRT scores as the performance measure.

The finding that the LRT scores decreased over the course of the mid-term assessment which aligns with our initial hypothesis is supported by studies (43–47) that showed decreases in CMJ height following handball-specific workload. As our mid-term assessments were executed on three consecutive training days, it can be assumed that the accumulated workload of the three sessions may have led to the decreased jump height. This finding also aligns with the conclusions drawn in the reviews by Claudino et al. (23) and Alba-Jiménez et al. (22), indicating that the validity and reliability of CMJs in detecting fatigue remain consistent over extended temporal periods. This indicates that factors that were not immediately detrimental (acute postexercise effect in the short-term assessment) on CMJ performance (i.e., LRT scores) might have had a negative effect in the mid-term (e.g., through metabolic by-products or reduced contractile function). This would correspond to various explanations of fatigue, such as reduction of sarcolemma excitability, restriction of cross-bridges, or increased effort perception (19) which may all need an incubation period before impacting performance. Therefore, the underlying mechanisms and detrimental factors in the performance-oriented LRT scores may differ between short-term and mid-term assessments. The results indicate that the LRT may be effective in detecting accumulated fatigue, specifically mid-term fatigue resulting from cumulative workload but not short-term fatigue.

In contrast to the findings of the LRT, we were able to detect an increase in perceived fatigue already at short-term (immediately after one training session), which clearly demonstrated that the LRT was not suitable for assessing objective fatigue after one training session. In support of our observation that fatigue can be assessed using subjective measures in handball players, Clemente et al. (64) reported that weeks with a high number of matches led to higher perceived fatigue and stress in 20 male professional handball players (mean age: 26.5 ± 4.9 years) who participated in the European Handball Federation Cup. Therefore, ASRM appear to be a good representation of all external and internal effects that an individual incorporates into his or her perceived state (26). Both ASRM detected fatigue during the short- (KEB) and mid-term (PRSS) assessment. However, if a detailed assessment of perceived fatigue is needed, more extensive and complex multiple-item questionnaires could be used to differentiate the underlying causes, such as stress, exhaustion, or illness.

### 4.2 Interaction between LRT and subjective measures of fatigue during short-, mid-, and long-term assessments

Contrary to the second hypothesis assuming interactions between LRT performance and ASRM (i.e., a decrease in LRT scores correlates with an increase in KEB or PRSS scores), we were unable to establish a general link between the LRT and the subjective assessment of fatigue, neither in the short-term nor in mid- or long-term. The only exception was a significant interaction effect between PRSS scores and workload on LRT scores in the long-term assessments. This interplay was not present for workload on LRT scores alone. Additionally, no direct or interaction effects were observed between ASRM, test time points, or workload on LRT scores both during the short- and the mid-term assessments. There may be several reasons for the lack of associations between the LRT and the ASRM in detecting short- and mid-term fatigue. The lack of short-term associations may result from the (contrary to our expectation) non-existent decrease in LRT scores immediately after the training while at the same time the subjectively perceived fatigue (as expected) increased (KEB score). Additionally, players may habitually expect a higher level of fatigue after training and subsequently report it (ASRM), even if it is not (yet) objectively present via LRT scores. Potentially, there was also only a low level of fatigue following the training sessions (in the short-term assessments) which may not be detectable in the LRT. In contrast and as proposed by Lombard et al. (65) low level fatigue may be detectable by subjective assessments leading to missing short-term correlations between the LRT and the ASRM in our sample. With regard to the absence of mid-term interactions, it can be postulated that the underlying mechanisms of both tests during a period of insufficient recovery (i.e., several consecutive training sessions on the following day) may be distinct. This suggests that the LRT is primarily performance-oriented and influenced by declines in neuromuscular performance, whereas ASRM are a complex compound score that is influenced by a multitude of psycho-physical factors. The physical and informational (decision-making) demands of handball are highly diverse (66). It is conceivable that a test that focuses exclusively on physical performance, such as the LRT, may prove to be less reliable in the context of team sports, which are also cognitive demanding. More specifically, team sports involve a higher degree of interaction with teammates and opponents, as well as a greater number of decisions, which may have an impact on informational capabilities that are not assessed in a jumping performance-related test. Consequently, the LRT may be less suitable for detecting the underlying patterns of handball-specific fatigue. This distinction between the two methods (subjective assessments being consistent in detecting perceived fatigue both during short- and mid-term fatigue and objective measures only detecting mid-term fatigue) is a key finding of the present study.

With respect to the detected long-term associations, it may be hypothesized that these mechanisms are different when assessments are undertaken every 2–4 weeks and recovery between the training sessions becomes more extended and the influence of the overall handball-specific and life load plays a more decisive role. Our results (in the case of the non-existent associations of objective and subjective fatigue in the long-term when workload was not included) confirm Buchheit (31) who reported that questionnaire items on (subjectively) perceived fatigue did not correlate with actual (objective) performance deteriorations in CMJ height in highly trained young handball players. The results additionally are in line with the results of Saw et al. (67) who in their review concluded that there is only a small correlation between objective and subjective fatigue parameters. However, when workload was included into the equation (i.e., the interplay of PRSS and workload on LRT scores) a significant interplay between subjective and objective fatigue was present. The three exemplary marginal slopes in Figure 2 demonstrate the interaction between workload and PRSS on LRT scores. This interaction indicates that at lower workloads, there is no apparent relationship between PRSS and LRT scores. However, with higher workloads, a more pronounced negative relationship between PRSS and LRT scores can be observed. This finding is noteworthy as it suggests that the training volume over weeks could be the decisive factor in bringing performance indicators and subjective assessments of fatigue to a common denominator. The result that higher previous workload leads to higher perceived fatigue goes in line with the review of Bestwick-Stevenson et al. (68) and McGuigan (1) who both reported that a higher workload leads to prolonged and increased fatigue. Additionally, Clemente et al. (64) reported that higher workload weeks increased perceived fatigue in 20 male professional handball players who participated in international club competitions.

### 4.3 Implications

Regarding the implications of the present results, a challenge of team training is that the training schedule is usually fixed for all players as a group, which limits flexibility of training times and days. It is additionally rather difficult to individualize and create different loads within team training, especially if the training takes place more collectively in game forms as it is often the case in handball. However, for additive strength and conditioning training, such an approach seems to be more practical as load parameters, are easier to quantify during this kind of training and can therefore be adapted according to individual levels of fatigue. Although objective procedures (LRT scores) demonstrated a correlation with subjective assessments (PRSS) in relation to workload in the long-term analysis, a combination of objective and subjective assessments remains a useful tool as according to Saw et al. (67) purely subjective assessments represent a valuable, however unspecific compound score. In contrast, the additional, primarily motor performance-oriented LRT allows practitioners to draw conclusions on whether the fatigue is more likely of neuromuscular origin (CMJ height) or due to the more subjectively rated compound score of perceived fatigue as being tested through the ASRM. This is also in line with Bourdon et al.'s (15) recommendation that it is advisable to combine the two measures of fatigue over time to assess training effectiveness and modify programs. This means that coaches and practitioners should consider the athlete's perceived physical condition, in addition to their performance fatigue. If the primary outcome of interest is categorized as a decrease in work capacity and the incapacity to produce the necessary muscle power to maintain simple or complex tasks, as defined by Enoka and Duchateau (59) and Taylor et al. (69), a test such as the LRT needs to be incorporated. This is consistent with Behrens et al.'s (19) definition of perceived fatigue which can exist without being able to measure neuromuscular performance fatigue (i.e., lower jump height). If, for example, it has been assessed that it is primarily neuromuscular aspects that cause fatigue (i.e., there are higher performance decreases in LRT scores compared to ASRM), the recovery-related measures or training adjustments should also be aimed in this direction (70). In case performance-oriented fatigue is lower than perceived fatigue, potential life load factors should be considered (71). In this regard, McGuigan (1) proposed to also individualize the recovery strategies.

### 4.4 Limitations

Several limitations of the present study need to be addressed. The study solely assessed quantitative external load, i.e., workload in hours. More precisely, in the present approach the intensity performed by the players in each training session or game is not known. Varying groups of players (distribution of female and male players as well as young adult and youth players) performed the individual test phases. Thus, the current results and inferences should only serve as supplementary indications that can complement the perception and observation of coaches (13) and cannot be generalized to other populations. Future studies should include an examination of the distinction between different performance levels (Tier 0–5) and between genders, with the aim of assessing whether the present results are also evident in other performance groups or across genders.

## 5 Conclusion

This work provides novel insight into (a) the immediate response to a training stimulus (short-term assessments), (b) the influence of a specific training week (microcycle), i.e., adaptation and recovery after a match followed by preparation for the next match (mid-term assessments), and (c) over an entire competitive season (macrocycle), with its typical progression of alternating recovery and adaptation phases to the high number of matches, with the aim of assessing chronic cumulative load adaptation (long-term assessments). For the first time, our results highlight that the ASRM items capture cumulative fatigue in handball players after short- and mid-term periods while in comparison, the LRT only detects fatigue in the mid-term but not for the short-term period. Furthermore, the lack of interaction between the objective and subjective fatigue measures during the short- and mid-term application indicates that the two measures do not depict the same components of fatigue and consequently should be used in combination to provide a more holistic picture of the level of fatigue during longer training periods. The significant interaction between PRSS score and workload on LRT scores suggests that the higher the workload, the greater the (negative) interaction between subjective (PRSS score) and objective (LRT score) fatigue measures. This implies that if handball players experience a high workload (≤10 h per week) assessing only one parameter (PRSS or LRT score) may be sufficient to detect fatigue.

## Data Availability

The raw data supporting the conclusions of this article will be made available by the authors, without undue reservation.
